# Comparison of three algorithms for estimating crop model parameters based on multi-source data: A case study using the CROPGRO-Soybean phenological model

**DOI:** 10.1371/journal.pone.0323927

**Published:** 2025-05-14

**Authors:** Yonghui Zhang, Yujie Zhang, Haiyan Jiang, Liang Tang, Xiaojun Liu, Weixing Cao, Yan Zhu

**Affiliations:** 1 School of Computer Engineering, Weifang University, Weifang, People's Republic of China; 2 Weifang People’s Hospital, Weifang, People's Republic of China; 3 College of Artificial Intelligence, Nanjing Agricultural University, Nanjing, People's Republic of China; 4 National Engineering and Technology Center of Information Agriculture, Nanjing Agricultural University, Nanjing, People's Republic of China; Jiangsu University, CHINA

## Abstract

Accurate prediction of crop phenological stage is essential for evaluating management strategies and assessing crop responses to environmental changes. In this work, we modified Non-dominated Sorting Genetic Algorithm with the core algorithm of PEST (MNSGA-II) and compared it to two other algorithms of Generalized Likelihood Uncertainty Estimation (GLUE) and Differential Evolution (DE) to calibrate the cultivar-specific parameters (CSPs) of CROPGRO-Soybean phenological model (CSPM) so as to exactly simulate the soybean phenology using the multi-source datasets of multi-site, multi-year, and multi-cultivar. Independent experimental data are used to validate the CSPM with the optimized parameters. The root means square error (RMSE), the mean absolute error (MAE), and coefficient of determination (R^2^) are used to evaluate the effects of different algorithms on calibrating the CSPs. The RMSEs (MAEs, R^2^) between all observed data and simulated data based on MNSGA-II, GLUE and DE are 4.28 (3.53, 0.9445) days, 4.76 (4.05, 0.9438) days and 5.17 (4.85, 0.9336) days, respectively, with little difference among the three algorithms. MNSGA-II has a certain advantage in calibration effect, and GLUE is the most stable during the repetition of each calibration. The MNSGA-II can be considered as a relatively ideal algorithm for estimating the crop model parameter. Which algorithm should be selected to calibrate the parameters of crop model according to the actual requirements. These results provide a reference to choose the suitable algorithm for estimating crop model parameter.

## 1. Introduction

Crop modeling is an important tool to study the impact of climate change on crop production [1]. Prediction of crop phenology is affected by model parameters, model inputs, and model structures. Parameter uncertainty in crop model mainly refers to the strong uncertainty in the values of crop cultivar-specific parameters (CSPs) and soil parameters. The uncertainty of model input data mainly includes the uncertainty of field observation data and meteorological data uncertainty. The structural design and complexity of the model vary greatly with different application purposes, and the structural uncertainty is one of the important sources of model uncertainty [2–3]. Uncertainty in parameter values is a vital factor in model prediction and has been a main research focus in reducing model uncertainty and improve model prediction [4–6]. CSPs are commonly used in crop models to quantify developmental features that differentiate between crop varieties, cultivars, or hybrids. However, because CSPs are unknown and hard to directly measure under field conditions, the estimation of CSPs is of great importance for obtaining reliable predictions using crop simulation models [7]. A number of methods have been developed to estimate crop model parameters, including trial-and-error [8–9], and automatic calibration [3,6,10]. Genetic algorithm (GA) is an efficient single-objective optimization algorithm [11]. Srinivas and Deb [12] adapted GA to solve the multi-objective problems, and developed a multi-objective algorithm known as Non-dominated Sorting Genetic Algorithm (NSGA), which was further improved as the NSGA-II by Deb et al. [13] NSGA-II has been widely applied in agriculture and hydrology fields [14–15]. Rodriguez et al. [14] used NSGA-II to identity best management means that effectively minimize nutrients pollution cost by providing optimal fronts between pollutant reduction and total net cost increase. Qie et al. [15] employed the NSGA-II to establish a model that could simultaneously optimize irrigation date and amount for saving water and increasing yield in the maize irrigation system.

The algorithm of GLUE is one of the most popular methods for estimating crop model parameters [6,16,17]. In combination with GLUE method, the quantitative influence of different combinations of the observed phenological stages on estimation of cultivar-specific parameters was explored by the CROPGRO-Soybean phenological model [18]. GLUE has been integrated into DSSAT system and is used to correct parameters for different crops, it is easy for users to operate and the performance of optimized parameters has been significantly improved [7].

The DE algorithm is a random search optimization algorithm using floating point vector encoding in continuous space. DE has the advantages of simple principle, few algorithm parameters and good search ability [19]. Some studies have shown that DE algorithm has good performance to estimate crop model parameters. Vesterstrom and Thomsen [20] employed DE, particle swarm and other evolutionary algorithms to 34 benchmark problems, the experimental results showed that DE had better performance than other algorithms. Zúñiga et al. [21] applied DE, covariance matrix adaptation evolution strategy, particle swarm and artificial bee colony to estimate the parameters of SUCROS growth model, and the results indicated that DE have the best optimization effect. Jiang et al. [22] improved the DE algorithm to calibrate the CSPs of rice phenology model with better estimating results.

Crop phenology determines the timing of various agronomic management measures. Accurate prediction of crop phenology is essential for evaluating management strategies and assessing crop responses to environmental and management changes [23–24]. He et al. [25] employed a Bayesian method to derive the parameters controlling canola flowering and maturity dates of APSIM-Canola model based on multi-source data of canola phenology in China. Liu et al. [26] used least squares parameter estimation to calibrate WheatGrow phenology model for four widely used cultivars in the main winter wheat production region of China.

Multiple algorithms have been utilized to optimize the parameters of crop models in recent studies to improve the simulated effects of the models. But the output of algorithm NSGA-II is a range of non-dominant solutions which is different to select optimal solutions for crop model parameter, thus NSGA-II has not been widely used to calibration parameters of crop models, and not been compared with other algorithms in optimization effects.

A multi-source experimental datasets was used for this study, including four soybean phenological stages of first flowering, first pod, first grain and first maturity stages in two years, two ecological sites, and nine soybean varieties. Using the CROPGRO-Soybean phenological model, the objectives of this study are (1) to modify the algorithm NSGA-II with the core algorithm of PEST to easily select the optimal solutions for crop model parameter, (2) to utilize the algorithms of MNSGA-II, GLUE, and DE to calibrate the CSPs (include CSDL, PPSEN, R1PPO, EM-FL, FL-SH, FL-SD, SD-PM) related to the simulation of phenological stages in CSPM and (3) to compare and analyze the calibration effects of the three algorithms. The algorithm selected by comparison with good performance would provide support to reduce the uncertainty for crop model.

## 2. Materials and methods

### 2.1 Brief introduction to CROPGRO-Soybean model

The model of CROPGRO-Soybean is a process-based model that simulates C, water, and N balances for the soybean plant and soil [27–28], which includes 15 cultivar parameters that characterize phenology and vegetative and reproductive growth. This study focuses the calibration of the three coefficients related to photoperiod sensitivity (CSDL, PPSEN, and R1PPO), and four coefficients related to photothermal duration of life phases (EM-FL, FL-SH, FL-SD, SD-PM) in CROPGRO-Soybean phenological model, which are associated with the simulation of the four phenological stages including the first flowering stage (FS), first pod stage, (PS) first grain stage (GS) and first maturity stage (MS). And these seven coefficients are usually calibrated in the cultivar level [29]. The value ranges typical for these parameters are described by Boote et al. [30], displayed in Table 1.

**Table 1:** The Cultivar-specific parameters to be calibrated in CSPM.

Symbols	Definition	Range
CSDL	the critical short day (h)	[11.78, 14.6]
PPSEN	the photoperiodic sensitivity (1/h)	[0.129, 0.385]
R1PPO	the increase in daylength sensitivity after anthesis (h)	[0.189, 0.549]
EM-FL	the ideal thermal day from seeding to bloom	[15.5, 28.9]
FL-SH	the time between first flower and first pod	[5.5, 10.0]
FL-SD	the time between first flower and first seed	[12.0, 16.0]
SD-PM	the time between first seed and physiological maturity	[12.0, 16.0]

### 2.2 Experimental design

The three field experiments (Exp. 1, Exp. 2, and Exp. 3) were conducted in two years of 2018 and 2019, and two sites of Nanjing in Jiangsu Province (32°3’32″N, 118°37’40″E) and Dangtu in Anhui Province (31°34’15″N, 118°29’52″E), involving nine Yangtze-Huai soybean breeding line labeled as Ci (i = 1, 2, …, 8, 9). Row planting was implemented with a planting depth of 3 cm, a row spacing of 0.5 m, and a row length of 2 m, in three replicates. The detailed information regarding the field experiments was provided in Table 2.

**Table 2:** Detailed information of the field experiments.

Experiment	Sowing time	Cultivar-Specific	Location	Latitude and Longitude	Observed data of phenological stages
Exp. 1	July 5, 2018	Ci (i = 1, …, 9)	Dangtu of Anhui	31°34’15″N, 118°29’52″E	initial flowering (FS), initial pod (PS), initial grain (GS), initial maturity stage (MS)
Exp. 2	June 21, 2019	Ci (i = 1, …, 9)	Nanjing of Jiangsu	32°3’32″N, 118°37’40″E	FS, PS, GS, MS
Exp. 3	June 24, 2019	Ci (i = 1, …, 9)	Dangtu of Anhui	31°34’15″N, 118°29’52″E	FS, PS, GS, MS

Crop management practices were in accordance with the recommendations of the local agriculture department. During the field experiment, no obvious light, temperature, water, nutrition, pest, or disease stresses were observed during the crop growth seasons, and the yield of each soybean cultivar is normal in the field experiments. Daily meteorological data were downloaded from the meteorological information center of the State Meteorological Administration of China.

### 2.3 Optimization algorithms

**Fig 1:**
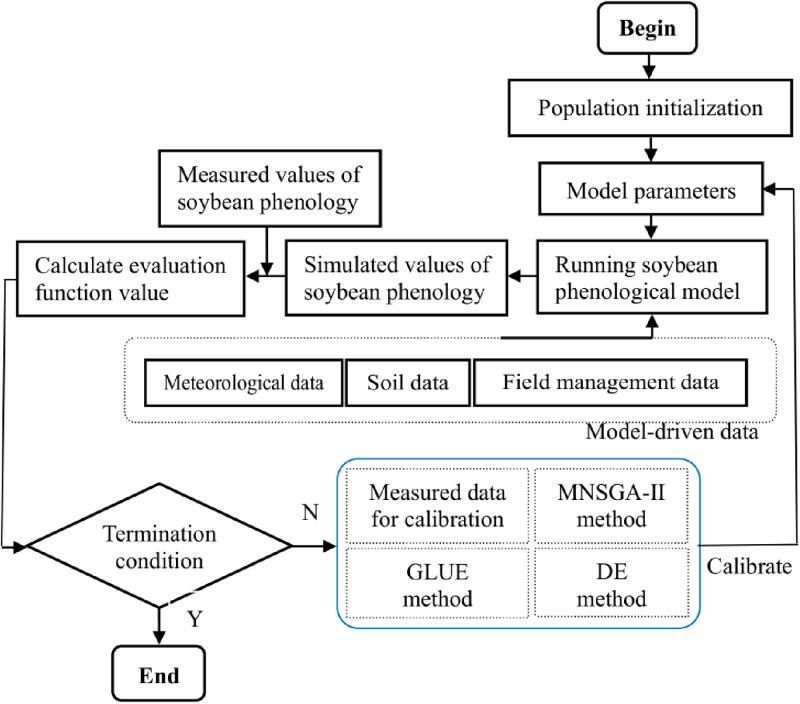
The framework of the three algorithms for estimating crop model parameters in this work.

According the framework in Fig 1, we employ three algorithms to calibrate the parameters of the CROPGRO-Soybean phenological model. The GLUE method was constructed by Beven and Binley [31], which was detailly summarized for calibrating the CSPM by Zhang et al. [18]. And other two algorithms are introduced as follows. The different algorithms are realized using Python programming language on IDE of Spyder in the Anaconda.

#### 2.3.1 The algorithm of MNSGA-II.

The main steps of the NSGA-II include initializing the parameter combinations, evaluating the combinations, fast non-dominated sorting process, calculating the crowding distance, two-chromosome tournament selection, crossover and mutation process, and combining the populations [13], described as follows.

Step 1: Initial parameter populations *P*_t_ is randomly generated using uniform distribution according to the ranges of parameters (Table 1).
Step 2: After non-dominated sorting for the initial populations of *P*_t_, the first-generation offspring populations of *Q*_t_ is obtained by the genetic algorithms with selection, mutation, and crossover.
Step 3: The paternal populations *P*_t_ and the offspring populations *Q*_t_ are merged as *R*_t_. After fast non-dominated sorting of *R*_t_, the crowding distance of each population in *R*_t_ is calculated.
Step 4: A two-chromosome tournament selection is used to select the best populations for the next generation. Two chromosomes in *R*_t_ are selected randomly and compared in terms of the front-rank and the crowding distance, the fittest populations are selected to form a new parental population.
Step 5: The new parental population is handled back to step 2, until the genetic generations have arrived.
Step 6: PEST is parameter-independent and uncertainty analysis-based software [32]. The core algorithm of PEST estimates model parameters by minimizing a given objective function of Eqn (1),

$$\phi ={\left(c-Xb\right)}^{t}M(c-Xb)$$ (1)

where X represents the action of a model under calibration conditions; b represents the parameter vector for this model; c is a vector of observations for which there are model-generated counterparts; and *M* is the cofactor matrix, a diagonal matrix in which the elements are the squares of the observation weights.

The NSGA-II needs to be modified to easily select the optimal solutions for crop model parameters. Since the output from NSGA-II is a non-dominant solution set composed of a certain number of individuals with optimized CSPs values, it requires a next step to select the optimal solutions from the non-dominant solution set. By integrating the NSGA-II with the core algorithm of PEST, we can attain the optimal parameter set, which is the optimized parameter combination with the non-dominated order of 1 that can minimize the *Err*_tol_ calculated by Eqn (2).


$$Err_{tol}=\sum_{i=1}^{N_{ps}}\omega_{i}\times abs(\frac{O_{i}-S_{i}}{O_{i}})$$ (2)

where *Err*_tol_ is the total error for the four phenological stages. *O*_i_ and *S*_i_ denote the observed and simulated values of phenological stages, respectively. *ω*_i_ denotes the weight coefficient, set as 1.0 in this work. *N*_ps_ is number of observed data of phenological stage, *N*_ps_ = 4 in this work. *abs*() denotes the absolute of a numerical value.

#### 2.3.2 The algorithm of DE.

The algorithm of DE is an optimization algorithm based on modern intelligence theory, which guides the optimization search direction through the swarm intelligence generated by the cooperation and competition among the individuals in a population [19]. The specific evolutionary process of DE is summarized as follows.

Step 1: According to the range of each optimized parameter (Table 1), uniform distribution is employed to randomly generate initial population of parameter combination.
Step 2: Fitness function of Eqn (3) is used to calculate the fitness value of each individual (parameter combination) in initial population to evaluate the simulated results of this individual.

$$FitFunc=\sqrt{\frac{1}{N_{ps}}\sum_{i=0}^{N_{ps}}{\left(O_{i}-S_{i}\right)}^{2}}$$ (3)

where *FitFunc* is the fitness function of DE. The meanings of other symbols are the same as above.

Step 3: If the evolution generation has arrived, the evolution process is terminated and the output solution in the last optimization round is taken as the optimal individual for the CSPM. Otherwise, the optimization continues.
Step 4: The intermediate population is generated by mutation and crossover operation for all individuals in initial population.
Step 5: Calculating the fitness values of each individual in intermediate population. A new generation population is obtained by evaluating the fitness of each individual in the initial and the intermediate population.
Step 6: The new generation of population is cycled back to Step 2.

### 2.4 Parameter settings for CSMP and the three algorithms

The required parameter value of the CSPM and the three algorithms are routinely set as follows:

The CSPM: Actual sowing depth, meteorological data and position latitude are input into CSPM, and the other required parameters for simulating the four phenological stages (FS, PS, GS, and MS) were used for the remaining parameters in CSPM.

The MNSGA-II: number of initial populations *N*_in_=1000, number of generation *N*_gn_ = 20, crossover probability(*pc*) parameter is 0.8, mutation probability (*pm*) is 1/*N*_op_, number of CSPs, *N*_op_ = 7, both the simulated binary crossover parameter and polynomial mutation parameter are set at a normal value of 20.

The GLUE: *N*_in_ =20000, *N*_gn_ = 1, the threshold value of likelihood function is set at 0.90.

The DE: *N*_in_ =500, *N*_gn_ = 40, parameter *pc* and *pm* are set at 0.5 and 2.38/(sqrt(2⊆*N*_op_)), respectively.

### 2.5 The evaluation criteria

Root mean square error (RMSE), mean absolute error (MAE), and coefficient of determination (R^2^) are used to evaluate the calibration effect of each algorithm, calculated by Eqn (4–6), respectively [18].


$$RMSE=\sqrt{\frac{1}{N_{\text{tol}}}\sum_{i=0}^{N_{tol}}{\left(O_{i}-S_{i}\right)}^{2}}$$ (4)

$$MAE=\frac{1}{N_{tol}}\sum_{i=1}^{N_{tol}}abs(O_{i}-S_{i})$$ (5)

$$R^{2}=1-\frac{RSS}{TSS}$$ (6)

where *N*_tol_ is the number of the observed or simulated values of phenology. RSS and TSS denote the residual sum of squares and the total sum of squares, respectively. All symbols used in this study are listed in Table 4.

**Table 3:** Data sources for calibration and evaluation.

ID	Calibration method	Calibration data	Evaluation data source
1	MNSGA-II, GLUE, DE	Exp. 1-Ci-FS, PS, GS, RS (i = 1, 2, 3)	Exp. 2-Ci-FS, PS, GS, RS (i = 1, 2, 3)
2	MNSGA-II, GLUE, DE	Exp. 2-Ci-FS, PS, GS, RS (i = 4, 5, 6)	Exp. 3-Ci-FS, PS, GS, RS (i = 4, 5, 6)
3	MNSGA-II, GLUE, DE	Exp. 3-Ci -FS, PS, GS, RS (i = 7, 8, 9)	Exp. 1-Ci-FS, PS, GS, RS (i = 7, 8, 9)

Exp.1-Ci-FS, PS, GS, RS (i = 1, 2, 3) denotes the observed data of phenological stages containing the first flowering stage (FS), first pod stage, (PS) first grain stage (GS) and first maturity stage (MS) from the cultivars of Ci (i = 1, 2, 3) in Exp. 1. The meanings other symbols are following this pattern.

**Table 4:** List of symbols used in this study.

Symbols	Meanings
*abs*()	the absolute value
CSP	the Cultivar-Specific Parameters
DE	the Differential Evolution algorithm
CSPM	the CROPGRO-Soybean phenological model
*Err*_tol_	the total error for the four phenology stages
*FitFunc*	the Fitness function of DE
GLUE	the Generalized Likelihood Uncertainty Estimation method
MAE	the mean absolute error
MNSGA-II	the modified Non-dominated Sorting Genetic Algorithm
*N*_fi_	the number of parameter combinations filtered by the likelihood function threshold value
*N*_gn_	the generation number of the optimization
*N*_in_	the scale of initial population
*N*_ps_	the number of observed phenology stages
*N*_op_	the number of the optimized variety parameters
*N*_tol_	the total number of the observed or simulated values
*O*_i_	the observed value of phenology stage
*pc*	the crossover probability
*pm*	the mutation probability
RMSE	the root means square error
R^2^	the coefficient of determination
RSS	the residual sum of squares
TSS	the total sum of squares
*S*_i_	the simulated value of phenology stage
*O*_*j*_	the observed value of the *j*^th^ output variable
*ω*_i_	the weight coefficient

### 2.6 The calibration and evaluation for the CSPs in CSPM

Each algorithm of MNSGA-II, GLUE, and DE and the observed phenological data of Ci (i = 1, 2, 3) in Exp. 1, Ci (i = 4, 5, 6) in Exp. 2, and Ci (i = 7, 8, 9) in Exp. 3 are utilized to calibrate the CSPs. The independent data of Exp. 2 (C1, C2, C3), Exp. 3 (C4, C5, C6), and Exp. 1 (C7, C8, C9) are used to evaluate the optimized CSPs. And the detailed information is described in Table 3. The calibration of each algorithm for one soybean cultivar is repeatedly executed three times, and three groups of optimized CSPs containing CSDL, PPSEN, R1PPO, EM-FL, FL-SH, FL-SD, SD-PM are generated. There are no significant differences among these three optimized CSPs using the T-test with the types of single tail and paired test (P > 0.05), the mean value of these three optimized CSPs is calculated as the optimal optimized value of the seven CSPs. The difference analysis results indicate these three algorithms are relatively stable in calibrating the CSPs of CSPM, and GLUE is the most stable during repeating each calibration.

## 3. Results

The RMSE, MAE, and R² were calculated using Eqn (4) and Eqn (6) to evaluate the calibration performance of the three algorithms: MNSGA-II, GLUE, and DE. The evaluation was conducted by comparing the calibration data from Exp. 1 with the evaluation data from Exp. 2, as well as the calibration data from Exp. 2 with the evaluation data from Exp. 3, and the calibration data from Exp. 3 with the evaluation data from Exp. 1.

**Fig 2:**
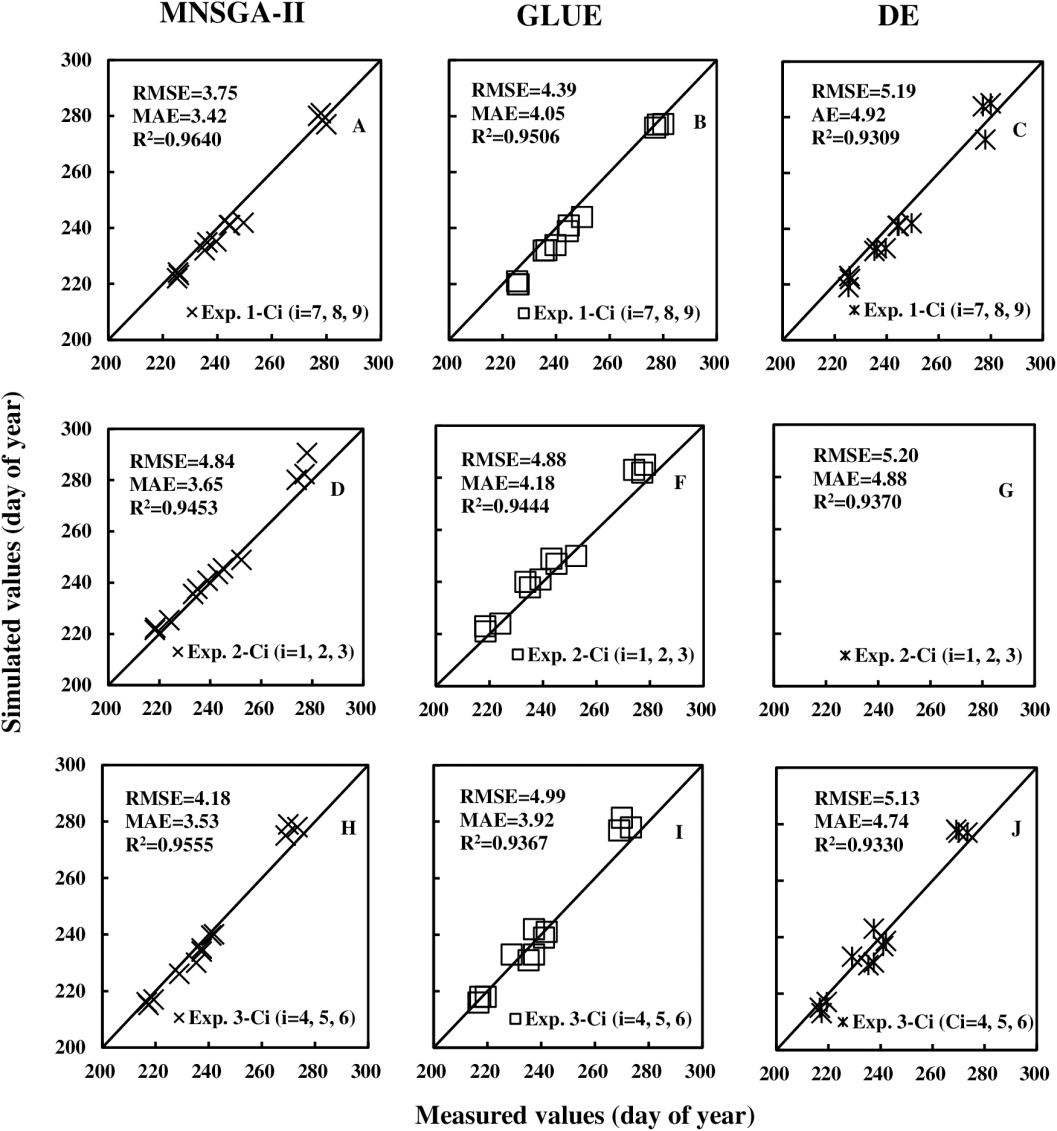
Comparisons of the measured and simulated data of phenological stages (day of year) using the different algorithms, calibration data, and evaluation data.

For the calibration data from Exp. 1 and evaluation data from Exp. 2, the calculated RMSEs (MAEs, R²) were 3.75 (3.42, 0.9640) days, 4.39 (4.05, 0.9506) days, and 5.19 (4.92, 0.9309) days for MNSGA-II, GLUE, and DE, respectively. Similarly, for the calibration data from Exp. 2 and evaluation data from Exp. 3, the RMSEs (MAEs, R²) were 4.84 (3.65, 0.9453) days, 4.88 (4.18, 0.9444) days, and 5.20 (4.88, 0.9370) days for the three algorithms. Additionally, for the calibration data from Exp. 3 and evaluation data from Exp. 1, the RMSEs (MAEs, R²) were 4.18 (3.53, 0.9555) days, 4.99 (3.92, 0.9367) days, and 5.13 (4.74, 0.9330) days, respectively. These results are visually summarized in the first, second, and third rows of Fig 2.

**Fig 3:**
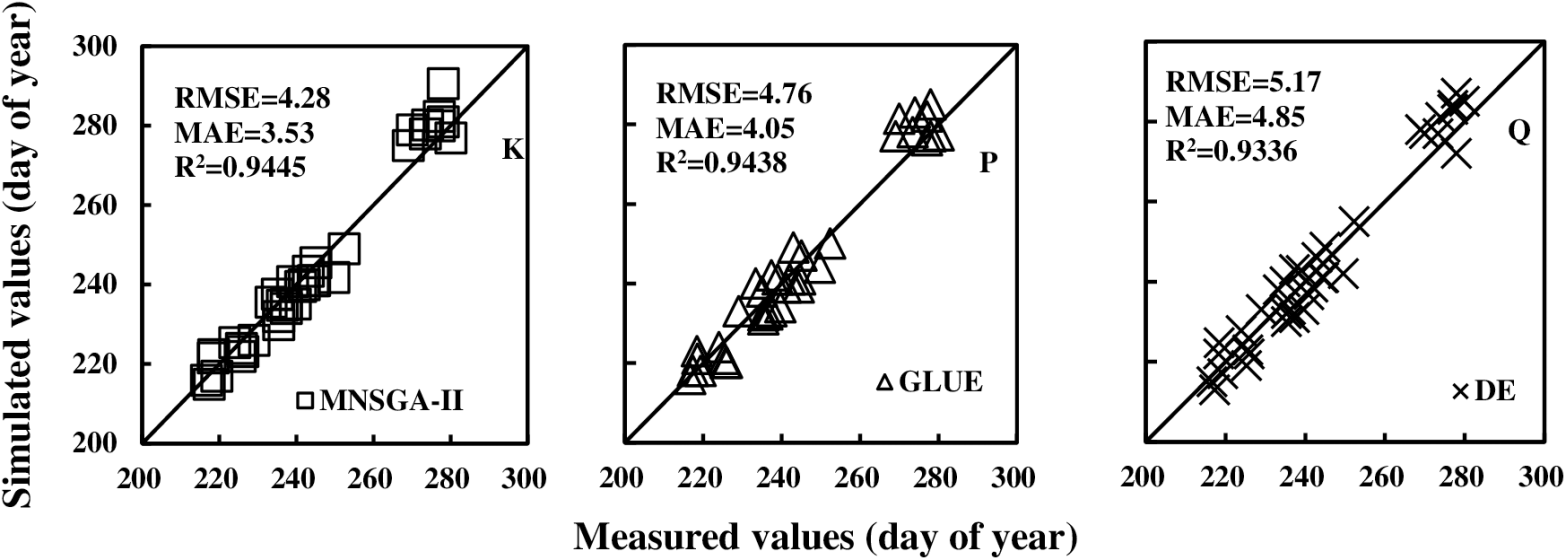
Comparisons of the measured and simulated data of phenological stages (day of year) using the different calibration algorithms.

Furthermore, the RMSEs (MAEs, R²) between all observed data and simulated data, based on the MNSGA-II, GLUE, and DE algorithms, were calculated as 4.28 (3.53, 0.9445) days, 4.76 (4.05, 0.9438) days, and 5.17 (4.85, 0.9336) days, respectively, as illustrated in Fig 3.

A comparative analysis of the RMSEs and MAEs reveals that the MNSGA-II algorithm exhibits a slight advantage over the GLUE and DE algorithms. However, the differences among the three algorithms are relatively minor, suggesting that all three methods perform comparably well in terms of calibration accuracy.

## 4. Discussion

In recent years, the parameters calibration of crop model has become a major way to reduce model uncertainty [10,11,22]. How to select a suitable algorithm to calibrate the parameters in crop models is a problem worth studying according to the actual requirement. However, there have been few comparisons among the different algorithms in estimating crop model parameters, most previous studies often focused on the parameters calibration of crop model using the single algorithm [7,25,26,33]. The algorithms of NSGA-II, GLUE, and DE have been applied in many fields due to the effectiveness and efficiency [7,15,21]. Based on multi-source observed data of soybean phenological stages, we conduct modification, comparison and analysis among these three algorithms of NSGA-II, GLUE, and DE to investigate the appropriate algorithm for more accurately estimating the CSPs in CSPM. NSGA-II algorithm outputs a non-dominant solution set in each genetic generation for estimating model parameters, selecting optimal solutions in the last generation through manual tests is required. In this work, we choose the optimal parameter set from a non-dominant solution set by integrating the NSGA-II and the core algorithm of PEST. This modification of the NSGA-II can optimize and simplify the selection of optimal solutions. Different likelihood functions of GLUE can generate different optimization results [16]. According to the characteristics of the observed phenological data, we choose the suitable likelihood function to ensure the calibration effect of GLUE on estimating CSPs. The threshold value is an important parameter in the likelihood function for choosing the optimal solution [34]. By a comparison, threshold value is set at 0.90 in this work. Meanwhile, the selected evaluation function of Eqn (3) obtains good results for DE to estimate model parameters. These measures above can ensure the calibration effects of the three algorithms.

T-test shows that there are no significant differences among the three parameter combinations obtained by repeating the calibration using a certain algorithm (P > 0.05). This demonstrates the three algorithms is stable for estimating the CSPs. However, the optimization algorithm itself has a certain volatility [10], not every simulated phenological stage could simultaneously arrive at a better result at the same time using a given algorithm. This phenomenon is also observed in our research. The calibration effects of considering the same algorithm for different soybean cultivars, different algorithms for the same cultivar, and even the same algorithm for the same cultivar often fluctuate (Fig 2). NSGA-II algorithm has rarely been used to estimate the parameters of crop model, but the MNSGA-II performs well in this study, therefore, it can be considered to calibrate crop model parameter.

we solely focus on the comparison and analysis of various algorithms to calibrate the CSPs of CSPM. Regrettably, we have not taken into consideration the stress conditions during the soybean experiments. Another limitation exists just taking CSMP as research object in this study. In later work, we will extend the optimization algorithms to crop models under stress conditions, and employ more target data including crop leaf area index, crop yield to verify the effects of different algorithms on calibrating the parameters of different crop models.

## 5. Conclusions

This study modified NSGA-II with the core algorithm of PEST, and calibrates the CSPs of the CSPM using three algorithms (MNSGA-II, GLUE, and DE) with multi-source datasets. Analysis revealed that all three algorithms exhibit stable parameter estimation, with GLUE showing the highest stability across repeated calibrations. While MNSGA-II slightly outperformed others in calibration accuracy (assessed via RMSE, MEA, and R²), differences among the three algorithms were marginal. Overall, MNSGA-II emerges as the most suitable algorithm for crop model parameter estimation. However, its generalizability to other crops requires further validation. In the further, we will improve and optimize the MNSGA-II to provide more efficient and accurate optimization of the crop model parameters.

## Supporting information

S1 File The data used in the figures. (XLSX)
